# Supplementary material for: The public health sector supply chain costs in Tanzania
Source: PLOS Glob Public Health. 2022 Nov 28;2(11):e0000960. doi: 10.1371/journal.pgph.0000960 (PMC10022133; doi:10.1371/journal.pgph.0000960)
Supplement: Attachment — Submitted filename: Reviewer comments_1&2.docx [file pgph.0000960.s001.docx]

| **Reviewer 1** | | | |
| --- | --- | --- | --- |
| **SN.** | **Reviewers Comments** | **Response** | **Page** |
|  | **GENERAL COMMENTS:** | | |
| 1. | However, I would have liked to see more clarity from the authors in the Discussion and specifically in terms of policy implications. The authors argue that “identifying the cost of operating the supply chain and the available financing options is vital to facilitate adequate resource allocation for the supply chain.”(rows 80-81). The question then needs to be: do the costs presented here reflect a well-functioning supply chain, or not? How will this study help decision makers and planners? Overall the Discussion section would benefit from being more clearly structured. Right now it has many threads, and it is not very clear to the reader what are the main points that the authors which to convey. | Thanks, the discussion section has been thoroughly edited for clarity. | **Pg. 11** |
| 2. | While the authors claim that the cost per unit would drop if more commodities were to flow through the system, clearly the total cost will increase. If there are budget constraints, how would the authors suggest these be approached? The authors attempt to make a few suggestions on how the current budget can be used more efficiently - such as just-in-time, 3PL, and resource allocation formula. It would be helpful if these suggestions could be grouped and presented more clearly to the reader in relation to the different cost elements. When the authors suggest developing a resource allocation formula (row 270), what exactly would they propose? Can they point to examples in the literature? | Thanks, this section has been restructured to ensure the discussion is focused on the study results | **Pg.11** |
| 3. | In terms of the proposal for a “just in time” model, it would be interesting to see the authors reflecting on the covid19 experience and whether this had any implications for Tanzania’s supply chain, and specifically whether a “just-in-time” model would be a less optimal solution during a global crisis when global supply chains are disrupted | Thanks, since the study used data before covid19 epidemic, we have restructured the discussion but didn’t discuss covid19 to avoid expanding the study beyond its scope. | **Pg 11** |
| 4. | Finally, the last two paragraphs in the Discussion would actually fit better in the introduction. It would also be helpful to add information upfront in the paper on what % share of the health system is public. (row 287: “the majority of the Tanzanian population” – can actual numbers be added?) | Thanks, the paragraph has been omitted | **Pg 11** |
|  | **SPECIFIC COMMENTS ON METHODS, RESULTS AND TABLES:** | | |
| 1. | Are costs presented here economic or financial costs? The methods mention both. | Thanks, the paper presents total cost that include financial and economic cost |  |
| 2. | Table 1 should be expanded to include a column showing the number of facilities per level. | Thanks, the number of facilities per level has been added | **Pg.5** |
| 3. | Table 4: how did you define the cost of serving clients? Please describe more clearly what this refers to. | Thanks, detailed explanation of the cost centres has been added…. “The resource was identified based on the core functions of the supply chain; i) procurement, included, time spent on identifying reliable supplies of good quality, management of bids and tenders and execution of procurement contracts. ii) Storage, included all resources utilized in receiving and warehousing of health commodities. iii) Distribution, included costs incurred in the movement of health commodities from central store to zonal warehouses and zonal warehouses to health facilities. iv) Management support, included all costs incurred in supervising and coordination of all supply chain activities, v) Logistics Management Unit (LMU), all the costs incurred in conducting LMU activities at MSD zones were costed. vi) Serving patients, these included all costs incurred to dispense health commodities to the clients….” | **Pg. 7** |
| 4. | Coherence of tables 3 and 4. It is unclear how the column headings in table 3 map to the rows/functions in table 4. Can the authors explain this in the paper? | Thanks, a column on total costs had been added to improve coherence of the two tables | **Pg.9** |
| 5. | Generally, the building costs look really high. Is this because of a 16% discount rate? Are the buildings newly constructed? This high cost should be explained and discussed in the paper. | Thanks, the key issue could be that the warehouse are currently not fully utilized…. A statement has been added in the discussion section… “Similarly, large and half empty warehouses provides an area for further efficiency improvement through increasing stock levels…” | **Pg.11** |
|  | **SPECIFIC COMMENTS ON EDITING** | | |
| 1. | Title of the manuscript: The title of the paper could be made more specific to inform readers: suggest to add the words “health sector” and “public/government” to the title. | Thanks, the new title reads ... The public health sector supply chain costs in Tanzania | **Title Pg.** |
| 2. | References: Some references are incomplete as they do not contain information on the publishers. – for example reference (8) is a USAID document and this should be added to the reference information. | Thanks, the reference has been updated to include the document publisher, date accessed and URL | **Pg 15** |
| 3. | Moreover reference (8) does not seem to be quoted properly as the text cites a commodity cost mark-up of 10-50% of value, and not 50% as written here by the authors (row 77 in the draft manuscript). (see https://www.ghsupplychain.org/sites/default/files/2019-07/financing_service_fees.cfm_.pdf ) Here I would therefore suggest that the authors change their text to read 10-50% or reword as “…up to 50%). | Thanks, the quote to the refence 8 now is reference 6 has been updated now reads… are estimated to be about 10% up to 50% of the value of procured commodities… | **Pg.3** |
| 4. | I have a similar comment on editing for reference 13 which is another USAID document and which has a recommended citation: “McCord, Joseph, Marie Tien, and David Sarley. 2013. Guide to Public Health Supply Chain Costing: A Basic Methodology. Arlington, Va.: USAID \| DELIVER PROJECT, Task Order 4.” It is unclear why the authors have not used the recommended citation. | Thanks, the reference has been updated to the recommended citation | **Pg. 15** |

**Reviewer 2**

| **SN.** | **Reviewers Comments** | **Response** | **Page** |
| --- | --- | --- | --- |
|  | **Abstract** | | |
| 1. | The methods section should provide brief information on the data sources for the analysis. It should also mention the levels of the health system the costing was done. And also please mention the types of costs presented (financial or economic costs). | Thanks, that text has been updated to add the levels of health systems….and indicated that the cost was both economic and financial… “four levels of the Tanzanian supply chain system were examined to determine the cost of running the supply chain by function. These included first the National level, second, Medical Stores Department (MSD) third, regional level, and fourth, Primary Health Care. The review adopted the program perspective, whereby all resources consumed in delivering health commodities were considered, resource use was then classified as financial and economic costs***…”*** | **Pg. 2** |
| 2. | There is a typo in line 45 which says two financials was. There are several other typos in the manuscript which should be corrected. | Thanks, it now reads…...The costing period was an average of two financial years, 2015/16 and 2016/17…. | **Pg. 2** |
| 3. | It is not clear how the determination that the operations are sub-optimal or inefficient as alluded to in line 49 and 51 was made. | A reference has been added to reflect on this quote, that the order fill rate was only 56% during the study period. | **Pg 12.** |
| 4. | The conclusion introduces new findings of information not provided in the results. New findings should not be presented in the conclusion. | Thanks, the discussion has been thoroughly edited to ensure consistency | **Pg. 11** |
|  | **Methods** | | |
| 1. | Line 98: It is not clear exactly what stratified purposive convenient sampling is. | Thanks, the term purposive convenient… has been replaced by non-probability … now reads.. ***“The multistage stratified non-probability sampling was employed.”*** | **Pg. 4** |
| 2. | Table 1 is not very helpful as readers who don’t know the Tanzania health system wont know any of those names or their significance or location. Why where those facilities selected? How was the sample size determined?What supply chain are conducted at each of those levels? | Thanks, the table has been, moved above just after the sub paragraph sample selection….the sample size was determine on non-probability sampling based on the discussion with Ministry of Health, President office Regional and Local Government Authority (PORALG) and MSD Facilities were selected  taking into consideration   - geographical location (urban/rural) - level of services offered - the feasibility of coverage within the given time constraints.   Functions of each level has been summarized in Figure 1 | **Pg. 5**  **Pg. 4** |
| 3. | The authors mentioned they used data from budgets and financial expenditures. Which data were from budgets and which ones were from financial reports? When both data existed, how were differences reconciled? | Thanks, it’s a typo the correct word is the expenditure report…This has been rectified. | **Pg.6** |
| 4. | The methods section is generally weak and it is not clear what exactly was costed and where the data for each were obtained from or the methods for the data analysis. | The method section has been extensively edited and reorganized to improve clarity. | **Pg. 5-7** |
| 5. | How were costs for shared resources allocated? | Thanks, a statement has been added on how we allocated shared resources “……. The proportion of the number of staff at each of the cost centre as a percentage of total staff at each of the study site i.e. central and zonal MSD, as well as health facilities to allocate shared resources such as building space, supplies, building operations costs etc.….” | **Pg.7** |
|  | **Results** | | |
| 5. | What is included in procurement costs – are commodity costs included or excluded? | Thanks, the costs included in the procurement has been explained in the method section…. procurement, included, time spent on identifying reliable supplies of good quality, management of bids and tenders and execution of procurement contracts... | **Pg 7** |
| 6. | In the results tables, distribution costs include buildings. Please explain what building costs are included in the distribution function? Again these things should have been clarified in the methods. | Thanks, this has been elaborated in the method section….” Distribution, included costs incurred in the movement of health commodities from central store to zonal warehouses and zonal warehouses to health facilities, and office spaces used by the distribution staff and vehicle garages…. | **Pg.7** |
| 7. | The tables should include sample sizes and also be clear that these are economic costs (if they are). | Thanks, the Sample size and the word economic and financial costs has been added… |  |
| 8. | The logistics management unit costs are presented for zonal medical stores but not for the central medical store. Why is this so? | Thanks, The logistics management unit are only at the zonal medical stores, further elaboration has been added in the introduction section….To improve performance of MSD and supply chain in Tanzania, MoH established a special unit under the development partner support to assist in organizing, monitoring, and supporting all supply chain activities for all health commodities at MoH and MSD zones | **Pg 3** |
| 9. | Table 4 shows total costs of sales. What is being sold by the medical stores? | Thanks, MSD procures bulk health commodities and health facilities set budgets that is used in purchasing of health commodities from MSD .. a statement has been added to the introduction to clarify….  Health facilities allocate funds in their budget and purchase health commodities from MSD … | **Pg.3** |
| 10. | Line 183 mentions costs of holding expired commodities. The methods don’t explain how this was costed. Please add this detail to the methods. | Thanks, a statement has been added to elaborate how the costs of holding expire commodities were computed… “To determine the costs of holding the expired health commodities, we computed the allocation key to determine the costs for expired commodities, the total value of expired health commodities was divided to the total health commodities managed at each MSD level to determine the percentage of expired commodity to the total value of stock managed by MSD during the study period. During discussion with MSD management, they identified storage and management support as the cost centers incurring the costs of holding the expired commodities. The allocation key then used to determine the costs of holding expired health commodities….” | **Pg.8** |
|  | **Discussion** | | |
| 1. | The discussion section is weak. There are statements where the reader does not see the linkage to the results and some of them seem more like views of the authors rather than discussion backed by the findings presented. For example, line 196 to 198 seem political and targeted to some stakeholder and hence not objective. Another example of non-objective statements is line 226 – it is not obvious that 3PL are cost-cutting, there are studies that have shown them to be more expensive and that is part of the reason they are not implemented. | Thanks, the discussion section has been thoroughly edited for clarity | **Pg. 11** |
| 2. | It is not clear what running warehouses in the box means as alluded to on lines 206 to 209. | Thanks, the assumption regarding WIB has been clarified … | **Pg. 11 line 451-459** |
| 3. | Line 254 to 260 should be backed by benchmarks which validate the statement. There are very few citations in the discussion, and this is a weakness as statements should be backed by facts from other studies. | Thanks, the discussion has been streamlined and additional references added to improve clarity and readability | **Pg.11** |
| 4. | The discussion section should include a section with the limitations of the study. | A section on study limitation has been included | **Pg.13** |
