# Supplementary material for: The public health sector supply chain costs in Tanzania
Source: PLOS Glob Public Health. 2022 Nov 28;2(11):e0000960. doi: 10.1371/journal.pgph.0000960 (PMC10022133; doi:10.1371/journal.pgph.0000960)
Supplement: Attachment — Submitted filename: Reviewer and Editors comment.docx [file pgph.0000960.s002.docx]

| **SN.** | **Reviewers Comments** | **Response** | **Page** |
| --- | --- | --- | --- |
| **Background** | | | |
| 1. | Background section page 3 rows 85-87. There is a claim that despite studies reporting significant gains in improving the performance of the public sector supply chain, the availability of essential health commodities has continued to remain uneven. Could references be added to sustain this claim? | Thanks, a reference (Ewen M, Kaplan W, Gedif T, Justin-Temu M, Vialle-Valentin C, Mirza Z, et al. Prices and availability of locally produced and imported medicines in Ethiopia and Tanzania. Journal of Pharmaceutical Policy and Practice. 2017;10(1):7.) has been added | **Pg. 3** |
| **Method** | | | |
| 2. | Some lack of clarity on the methods on page 6: row 151 where the authors mention having used budget-based estimates, but the following sentence indicates that expenditure reports were used. Here there is lack of clarity. Did the study look at budgets (pre-expenditure), expenditure data (post-expenditure), or both? Additional clarification is needed here with more specific detail (expenditure reports at the level of facility, district? Etc) | Thanks, we used audited financial reports to obtain the throughput values, therefore the word ….. ***“Audited financial reports (post expenditure reports) ”*** Has been used to improve clarity. | **Pg.5** |
| 3. | page 7, rows 176-178: it is unclear what costs are included under category (vi) serving patients. Can the authors give concrete examples here. | Thanks a statement to highlight this costs has been added….. “such as salaries for health workers dispensing the health commodities, building space, furniture etc. ..” | **Pg. 6** |
| 4. | under resource measurement and valuation, page 7 row 180: “Resource use was classified as financial and economic cost”. However it appears that authors have only used one classification and valuation approach, and not a dual approach. In a dual approach financial and economic costs would be valued and reported separately, and their amounts would differ. Generally, financial costs are actual monetary expenditures for resources, such as staff salaries, facility rent, and medical supplies. The economic costs, on the other hand, are valued differently, according to their opportunity costs including those resources that are not paid for (but must be monetized), such as volunteer time, caregiver time, and donated space and equipment. Are the authors certain that the costs shown in the paper represent both economic and financial costs? Or is it rather just financial costs being presented here? | Thanks for pointing to this, description of the financial and economic costs have been added …. “the financial costs included all costs that are directly incurred by MSD or health facilities for health commodities delivery and the economic costs included all costs that are contribute in the delivery of health commodities but are not directly paid by MSD or health facilities such as donated goods and workers paid by development partners…”  are  Also included in table 2 and 3. Due to the size of the table we had presented them combined, but we concur with you presenting separately improved clarity. | **Pg. 6.**  **Table 2 and 3** |
|  |  | | |
|  | **RESULTS** | | |
| 1. | There is an issue with the overall numbering of tables: there are two tables labelled as Table 4. | Thanks, the numbering has been made consistent |  |
| 2. | Some additional work is needed by the authors to explain how the different tables link to each other. If I understand correctly, there is no overall results table for costs at all levels? Reading the first sentence in the Results section, I assume that the total costs for the public health sector supply chain (please use the same terminology as in the title paper here) is = 15.5 + 4.1 = 19.6 million? Is there a way that you could combine tables 2 and 3 and show this combined cost somewhere in a table? | Thanks a lot for your invaluable inputs. I concur that the results were somehow confusing. Therefore, the method section has been streamlined. The main result of the study is the average cost of running a supply chain at different levels of the supply chain. At the Medical Stores Department there is two parts Central level and Zonal level. The description or the architecture of the Tanzania public supply chain system has been further described in the manuscript. This were costed separately to be able to estimate the average cost at each level (The zonal level only four zones were sampled among the eight zones). The cost are not additive but provides average costs at each level. The next level was the health service level. The method and results have been edited to improve clarity. | **Pg.4 -11** |
| 3. | page 9 row 224: the value reported here (23%) does not appear in the table referred to. Suggest that the 23% value be added to Table 2. Or should it rather be added to table 4/5? | Thanks, the values have been added on table 2 | **Pg. 8** |
| 4. | table 4 (page 10): I would suggest to add one more column with Total values for the country (national /average values). This will make the paper more helpful for other researchers. I would assume the value would then be 23% in the bottom row (? See comment 7 above). However I am not clear on what data the tables actually present. | Thanks, A column has been added and a statement in the text… “  The value is 13.4% for the operation cost of the supply chain at MSD zonal level per value of the health commodities. The 22.2% is the average operation costs at the central MSD | **Pg.10** |
| 5. | Results: I would strongly suggest to remove the words “economic and financial (costs)” from all the table headings. The approach should be clear from the methods section | The terms financial and economic has been removed throughout the tables |  |
|  | **DISCUSSION** | | |
| 1. | Section, page 12, row 275: I would urge the authors to avoid sweeping statements like “For obvious reasons…” as the reasons are not obvious to the reader | Thanks, the statement has been edited now reads… … “the strategy is likely to reduce supply chain…” | **Pg.11** |
| 2. | Page 13, rows 307-309: “it may be that…… empty warehouses…..”. Can the authors please include evidence or references to evidence that warehouses are empty. | Thanks, the statement…..has been deleted. | **Pg 12** |
| 3. | Page 13, rows 313: “The findings of this study imply that the supply chain might be operating at suboptimal levels” – why? Could you please explain what are the specific findings that point to this? Is it linked to costs? | Thanks, the statement has been further reviewed and rewritten now reads…  The findings of this study imply that the supply chain system might be operating at suboptimal levels at lower lever facilities i.e. the primary health care, when examined on based on the average supply chain costs per unit of health commodities managed which was found to be 37% of the value of health commodities managed at this level….. …Apart from this high unit costs estimated, issues of poor quality of data pertaining value of health commodities procured/delivered at this level cannot be underscored as such these results should be interpreted with caution….. | **Pg.12** |
| 4. | Page 13, rows 319: the term “vertical program” requires more explanation as readers may not know what this means | Thanks…. “Vertical program commodities i.e., the health commodities for programs that are disease specific and mainly donor funded. These includes HIV/AIDS, Tuberculosis, Malaria and Family Planning commodities among the few | **Pg. 12** |
|  | **CONCLUSION** | | |
| 1. | The authors argue that the supply chain should be a target for cost reduction. I do not see how this conclusion was made based on data presented in the paper, as a 23% overhead is within the 10-50% range presented in the background section. Perhaps cost reduction is the wrong term, as it may lead to associations around budget cuts; and “efficiency gains” might be more appropriate terminology. | Thanks, the term “Cost reduction has been replaced with improve supply chain efficiency…..  “Increasing the quantities of items managed at the health facilities to meet the demand would significantly improve supply chain efficiency at this level of service” | **Pg.15** |
|  | **GENERAL COMMENT** | | |
| 1. | It seems that with the revision process some additional editing may be needed to check language and grammar carefully before making the final submission. | Extensive editing has been done throughout the manuscript. |  |

**Editors Comments**

| **SN.** | **Reviewers Comments** | **Response** |
| --- | --- | --- |
| 5. | Please review your reference list to ensure that it is complete and correct | The reference list has been reviewed |
| 6. | Please amend your detailed Financial Disclosure statement. This is published with the article. It must therefore be completed in full sentences and contain the exact wording you wish to be published | The sentence has been reviewed and it is correct |
| 7. | Please ensure that the Title in your manuscript file and the Title provided in your online submission form are the same | Thanks this is observed |
